# MACHINE LEARNING-BASED PREDICTION MODEL FOR IN-HOSPITAL MACCE IN OLDER PATIENTS WITH CHD

**DOI:** 10.1093/geroni/igae098.4021

**Published:** 2024-12-31

**Authors:** Wen Tang, Xuedong Wang, Xuebing Yang, Ying Sun

**Affiliations:** Beijing Friendship Hospital, Capital Medical University, Beijing, Beijing, China (People’s Republic); Hainan University, Haikou, Hainan, China (People’s Republic); Chinese Academy of Sciences, BEIJING, Beijing, China (People’s Republic); Beijing Friendship Hospital, Capital Medical University, BEIJING, Beijing, China (People’s Republic)

## Abstract

**Objectives:**

Older patients with coronary heart disease (CHD) are at a higher risk of major adverse cardiac and cerebrovascular events (MACCE) during hospitalization. This study aims to develop a risk prediction model for in-hospital MACCE events in older patients with CHD using emerging machine learning methods.

**Methods:**

The study included 885 older inpatients with CHD (74.68±6.89 years, 58.3% male). We created prediction models via XGBoost, Support Vector Machine(SVM), random forests, Histogram-Based Gradient Boosting Classifier(HGBC) and Gradient Boosting Decision Trees(GBDT) using 38 features, and evaluated its performance using five-fold cross-validation..

**Results:**

MACCE was identified in 106 patients. The XGBoost model demonstrated the best performance, achieving an accuracy (ACC) of 93.56% and an area under the curve (AUC) of 0.8736 (95% confidence interval: 0.756–0.967). The SVM model achieved an ACC of 90.17% and an AUC of 0.8028 (95% CI: 0.684–0.906); the Random Forest model achieved an ACC of 91.53% and an AUC of 0.8675 (95% CI: 0.764–0.953); the HGBC model achieved an ACC of 93.45% and an AUC of 0.8550 (95% CI: 0.741–0.953); and the GBDT model achieved an ACC of 93.11% and an AUC of 0.8674 (95% CI: 0.756–0.957). Finally, SHAP analysis revealed that the top three most important features were troponin T, the FRAIL score, and the NYHA functional classification.

**Conclusion:**

Machine learning models can effectively predict in-hospital MACCE events in older patients with CHD. Troponin levels, frailty status, and cardiac function are strong predictive indicators.

